# Participant valued appearance of bone conduction devices: a comparison between percutaneous and transcutaneous systems

**DOI:** 10.1007/s00405-025-09335-7

**Published:** 2025-03-22

**Authors:** Hidde K. Krijnen, Tjerk W. Aukema, Myrthe K.S. Hol

**Affiliations:** 1 Department of Otorhinolaryngology/Head and Neck Surgery, University Medical Center Groningen, PO Box 30.001, Groningen, 9700 RB The Netherlands; 2 Research School of Behavioral and Cognitive Neurosciences, Graduate School of Medical Sciences, University of Groningen, Groningen, The Netherlands; 3 Department of Otorhinolaryngology/Head and Neck Surgery, Radboud University Medical Center, Nijmegen, The Netherlands

**Keywords:** Bone conduction device, Patient preference, Appearance, Percutaneous, Active transcutaneous

## Abstract

**Purpose:**

To investigate whether the appearance of percutaneous bone conduction devices (perBCDs) or active transcutaneous bone conduction devices (atBCDs) is preferred by BCD-users and non-users. The second aim is to examine the degree to which the appearance of a device matters in comparison to other BCD traits, and whether certain participant characteristics predict this.

**Methods:**

An online questionnaire was designed and administered to BCD-users and non-users (i.e., persons with no experience using a BCD). Pair-wise comparisons showing pictures of the latest generation perBCD and atBCD sound processors and implant sites were anonymously provided to participants, who could indicate their preference. Sum scores were calculated ranging from − 2 (strong preference for perBCD) to 2 (strong preference for atBCD). Means for the total score as well as sub scores of pictures showing either sound processor or implant site were calculated. Statements were presented in which the appearance of the device was weighed against other traits such as better hearing.

**Results:**

The study population consisted of 102 BCD-users and 105 non-users. An overall preference for perBCD sound processors was observed (mean score − 0.50 (95% CI: -0.63, -0.37). BCD-users had no preference for implant sites whilst non-users preferred atBCDs (-0.03 (-0.27, 0.21) and 0.60 (0.40, 0.80) respectively, *p* < 0.01). Most participants found better hearing more important than having an appealing device (*n* = 150, 73.0%).

**Conclusions:**

PerBCD sound processors were preferred over atBCD sound processors by both BCD-users and non-users. Functionality seems to be more important than the appearance of the device.

**Supplementary Information:**

The online version contains supplementary material available at 10.1007/s00405-025-09335-7.

## Introduction

Bone conduction devices (BCDs) play a crucial role in hearing rehabilitation for patients with mixed or conductive hearing loss, and to a lesser extent, single-sided deafness (SSD) [1]. BCDs generate mechanical vibrations that either stimulate the bone directly (i.e., direct drive) or transmit vibrations to the skin and underlying tissue (i.e., skin drive) [2]. The applicability of passive transcutaneous skin drive BCDs is limited due to their inferior audiological performance compared to direct drive counterparts and the occurrence of skin complications such as pain or necrosis [3–4].

The first direct drive BCD, the percutaneous Bone Conduction Device (perBCD), was fitted by Tjellström et al. in 1977 [5]. The perBCD consists of an implanted osseointegrating screw in the temporal bone and a skin-penetrating abutment. Externally a sound processor is attached. In 2012, a new type of direct drive BCD was introduced: the active transcutaneous BCD (atBCD) [6]. An atBCD typically consists of an implant containing a receiver coil, transducer, and connecting magnet, with an externally attached sound processor that converts sound into an electromagnetic signal.

Both perBCDs and atBCDs have their respective advantages. PerBCDs can be used with higher bone conduction thresholds [7], have lower initial purchase costs [8], require less invasive surgery [2] and produce less bothersome artifacts on magnetic resonance imaging [9]. In contrast, some studies suggest that atBCDs offer a better safety profile concerning skin-related complications and implant loss [8, 10]. Another advantage of atBCDs is their lower susceptibility to feedback at higher frequencies [11]. Lastly, the appearance of transcutaneous systems is often deemed to be more appealing than percutaneous ones, primarily due to the absence of a skin-penetrating abutment [12–16].

To the best of our knowledge, no systematic studies have actually compared the physical appearance of perBCDs with transcutaneous systems. When retrospectively asked, both percutaneous and transcutaneous BCD users seem satisfied with the appearance of their device [17–19]. However, responses may be influenced by confirmation bias, where individuals justify past decisions [20]. Furthermore, persons who are bothered by the appearance may refrain from BCD use in the first place [13, 21, 22]. Regarding conventional hearing aids, persons tend to prefer the least visible device [23]. Congruously, in an effort to address hearing aid stigmatization, manufacturers have focused on developing increasingly smaller and less visible hearing aids [24]. This trend may also be observed with BCD sound processors. Yet, neither percutaneous nor transcutaneous systems are evidently less visible than its counterpart, making it difficult to compare the appearance of these BCDs in an objective manner.

Choosing for either perBCD or atBCD is an effortful decision for the patient from a medical, audiological, cultural and psychosocial perspective. During this decision making process, the patients’ values and preferences should play a central role [25–26]. This is achieved by making use of decision making techniques such as shared decision making. It has been demonstrated that shared decision making can enhance patient satisfaction and adherence to long-term treatments [27]. A crucial aspect of this process is providing objective counselling on the advantages and disadvantages of the treatment options. However, the lack of literature regarding perBCD and atBCD appearance currently impedes the provision of such unbiased patient information.

The primary aim of this study is to gain insight in the subjective differences between perBCDs and atBCDs by investigating whether the appearance of either perBCDs or atBCDs is preferred by both BCD users and non-users. However, insight into BCD appearance preference would only be valuable when placed into context. Previous studies indicate that certain individuals place greater value on, or are more bothered by the appearance of a BCD [13, 21, 22]. Therefore, the second aim of this study is to examine the degree to which the appearance of the device matters in comparison to other BCD traits, and whether certain participant characteristics predict this. Lastly, the newly designed questionnaire will be evaluated for applicability to a broader audience.

## Methods

### Study design

The current study was conducted in a tertiary hospital). The study is of cross-sectional nature. An online questionnaire was administered using the online software Qualtrics (Provo, Utah; https://www.qualtrics.com). Incorporation of pictures of BCD users as well as the distribution of the questionnaire was approved by the Central Ethics Committee of our hospital (register number 19404).

### Questionnaire design

The questionnaire was designed by two experts in the field along with two researchers and an epidemiologist specialized in psychometrics and health related outcome measurement (Supplementary Material A, English version of the questionnaire).

**Fig. 1:**
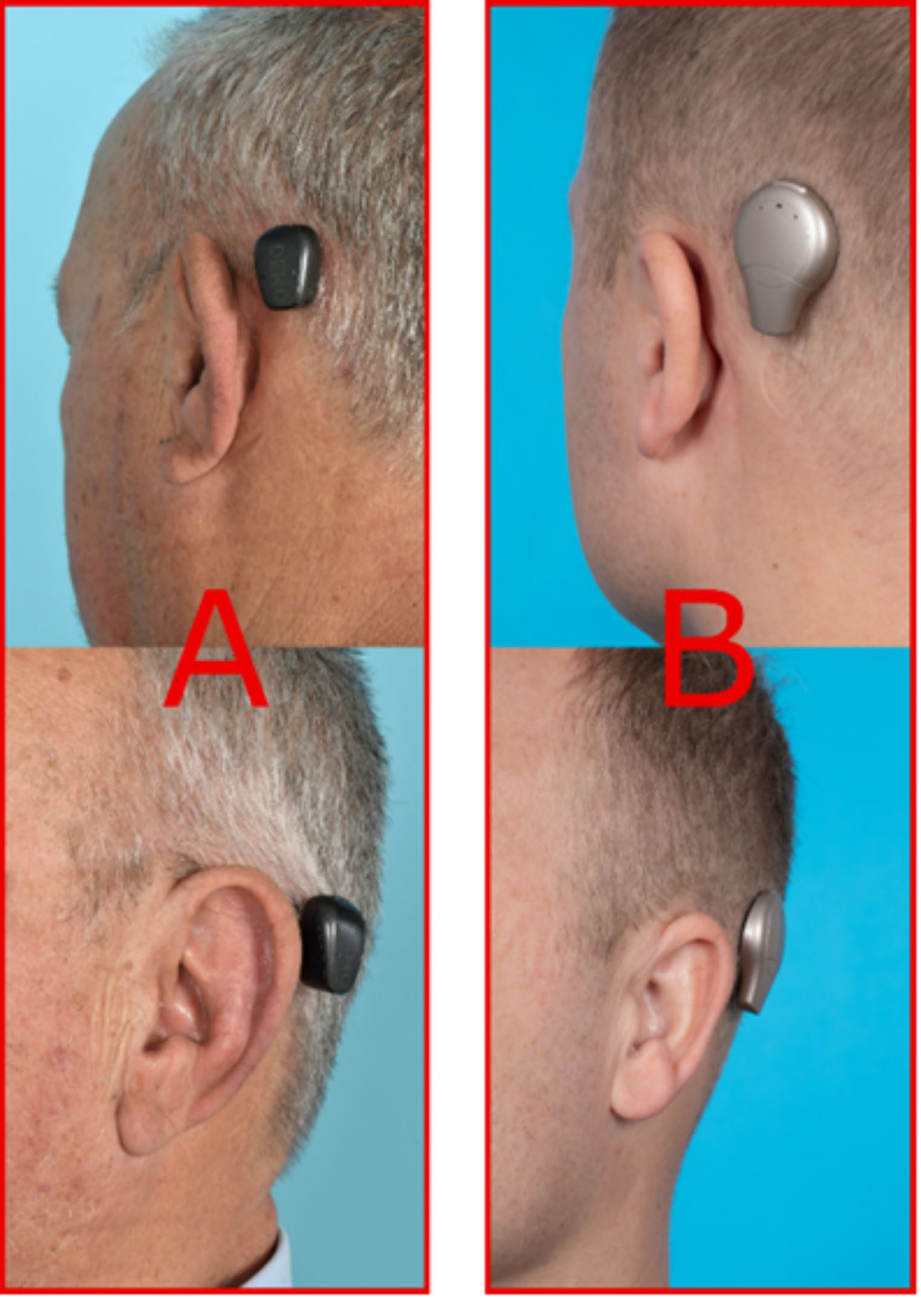
Example of a pair-wise comparison showing a perBCD (left, **A**) versus an atBCD (right, **B**) on models with short hair

The questionnaire consisted of four domains. The first domain involves participant characteristics. The second domain was designed to compare the physical appearance of perBCDs and atBCDs. Professional photographs were taken of both males with short hair and females with long hair wearing a perBCD (Baha 5 or 6 by Cochlear ltd., Sydney, Australia, or Ponto 5 Mini by Oticon Medical, Vallauris, France) or a atBCD (Osia 2 by Cochlear or Sentio by Oticon Medical). Photographs were taken with the sound processor attached as well as from the implant site. PerBCD and atBCD photographs were placed in a panel to allow for pair-wise comparison (Fig. 1).

Participants were instructed to indicate their preference on a 5-point Likert scale for either the left or right device for a total of 12 pair-wise comparisons in random order. A score of-2 represented a strong preference for the device on the left and 2 represented a strong preference for the device on the right.

In the third domain, respondents were instructed to pick the BCD model with the most preferrable appearance. Based on expert opinion and research group discussion, seven traits were identified that were weighed against the most preferrable BCD appearance. An example of such a statement is the following: ‘I would still choose the device with the most appealing appearance, even if my doctor would recommend a different device’. Participants were required to report their level of agreement on a 5-point Likert Scale ranging from − 2 (strongly disagree) to 2 (strongly agree). The forth domain of the questionnaire featured open questions, which are omitted from discussion for reasons of conciseness.

The questionnaire was primarily constructed in English to ensure future adoptability for the international public. Forward and backward translation was performed following the guidelines provided by Beaton et al. [28]. As the current questionnaire is not a validated instrument, the remaining translation steps were omitted.

### Participants

The questionnaire was administered to two groups of individuals. The first group consisted of individuals who either have used a BCD in the past or are currently using one, herein referred to as BCD users, from a tertiary university medical. The second group consisted of participants from the general population who have never used a BCD, referred to as non-users.

The inclusion criteria for both groups the group was an age of older than 16 years. No further criteria were formulated.

BCD users were approached by e-mail. Non-users were recruited using social media.

### Statistical analysis

All characteristics will be stratified into total group, BCD users and non-users and presented using appropriate statistics depending on data type). Differences between BCD users and non-users were statistically tested using either t-test, Kruskal-Wallis, Mann-Whitney U oror Chi-square.

All Likert scale preference ratings were transformed so that a score of -2 reflects a strong preference for perBCD and 2 a strong preference for atBCD. Mean scores were calculated for the total of 12 pair wise comparisons and for the sub scores of pictures showing BCD users with and without the sound processor attached and with long and short hair. All mean scores were tested against the null hypothesis of equals to zero using one sample t-test. The total and sub scores were presented with means and 95% confidence intervals. Further stratification for BCD-users and non-users was performed and scores were presented. Potential differences between BCD-users and non-users for the total and sub scores were tested by using two-sample t-tests, and the effect size was estimated using Cohen’s d.

For the importance of BCD appearance, the answers to the 5 point Likert scales were presented with frequencies in a diverging stacked bar chart. Further, a mean score was calculated and presented with mean and SD. Potential mean score differences based on participant characteristics will be tested with appropriate statistical tests.

The internal consistency reliability will be analyzed by means of Cronbach’s alpha. A Cronbach’s alpha of larger than 0.7 was considered acceptable. Improvement of the scale by more than 0.1 was considered a substantial improvement upon item deletion.

*P*-values of < 0.05 were considered to be statistically significant. In case of paired testing a Bonferroni correction was applied. All data was analyzed using IBM’s Statistical Package for the Social Sciences (SPSS) version 28. Visual representation of mean preference scores was performed using GraphPad Prism (GraphPad Software, San Diego, CA) version 10.2.3. Diverging stacked bar chart was created using R package version 4.4.0 (R Foundation for Statistical Computing; https://www.r-project.org).

## Results

In total, 274 participants were recruited (123 BCD users versus 151 non-users, response rate for BCD users 51.6%). After deletion of incomplete responses, responses with missing age and age of < 16 years, 207 participants remained (102 BCD users versus 105 non-users). Nine participants filled in the English version of the questionnaire, whilst 198 filled in the Dutch version. BCD users were significantly older than non-users (61.8 years versus 39.7 respectively, *p* < 0.01) (Table 1). Non-users were more frequently of male gender when compared to BCD users (64.8% versus 52.0% respectively), although this difference was marginally non-significant (*p* = 0.06). Most BCD users used a perBCD (93.8%) and used their BCD for 8–16 h per day (87.9%). Nine BCD users (8.8%) reported having an outer ear malformation.

**Table 1:** Participant characteristics stratified by BCD use

Variable	Total group (*n* = 207)	BCD users (*n* = 102) ^a^	Non-users (*n* = 105)	*P* value ^b^
Age (years), mean (SD)	50.6 (20.3)	61.8 (15.7)	39.7 (18.3)	< 0.01
Gender				0.06
Male	121, 58.5%	53, 52.0%	68, 64.8%	
Female	84, 40.6%	48, 47.1%	36, 34.3%	
Not specified	2, 1.0%	1, 1.0%	1, 1.0%	
Hair style (long)	75, 36.2%	32, 31.4%	43, 41.0%	0.15
Hearing loss (yes) ^c^	-	-	23, 21.9%	-
Hearing aid user (yes) ^c^	-	-	14, 13.3%	-
BCD knowledge (yes)	-	102, 100.0%	35, 33.3%	-
Quit using BCD (yes) ^d^		9, 8.8%	-	-
BCD type	-			
perBCD	-	90, 93.8%	-	
atBCD	-	3, 3.1%	-	
Other ^e^	-	3, 3,1%	-	
Daily hours of BCD use				
0–4 h	-	5, 5.5%	-	
4–8 h	-	6, 6.6%	-	
8–16 h	-	80, 87.9%	-	
Outer ear malformation (yes) ^c^	-	9, 8.8%	-	

BCD: bone conduction device; SD: standard deviation; perBCD: percutaneous BCD; atBCD: active transcutaneous BCD ^a^ BCD-users were defined as anyone who has used or currently uses a BCD; ^b^*p*-values of < 0.05 were considered statistically significant; ^c^ As reported by the participant; ^d^ BCD-users who do not use a BCD anymore; ^e^ Other types of BCD included Baha Attract (*n* = 2) and one person with a spectacle BCD

### BCD appearance preference

**Fig. 2:**
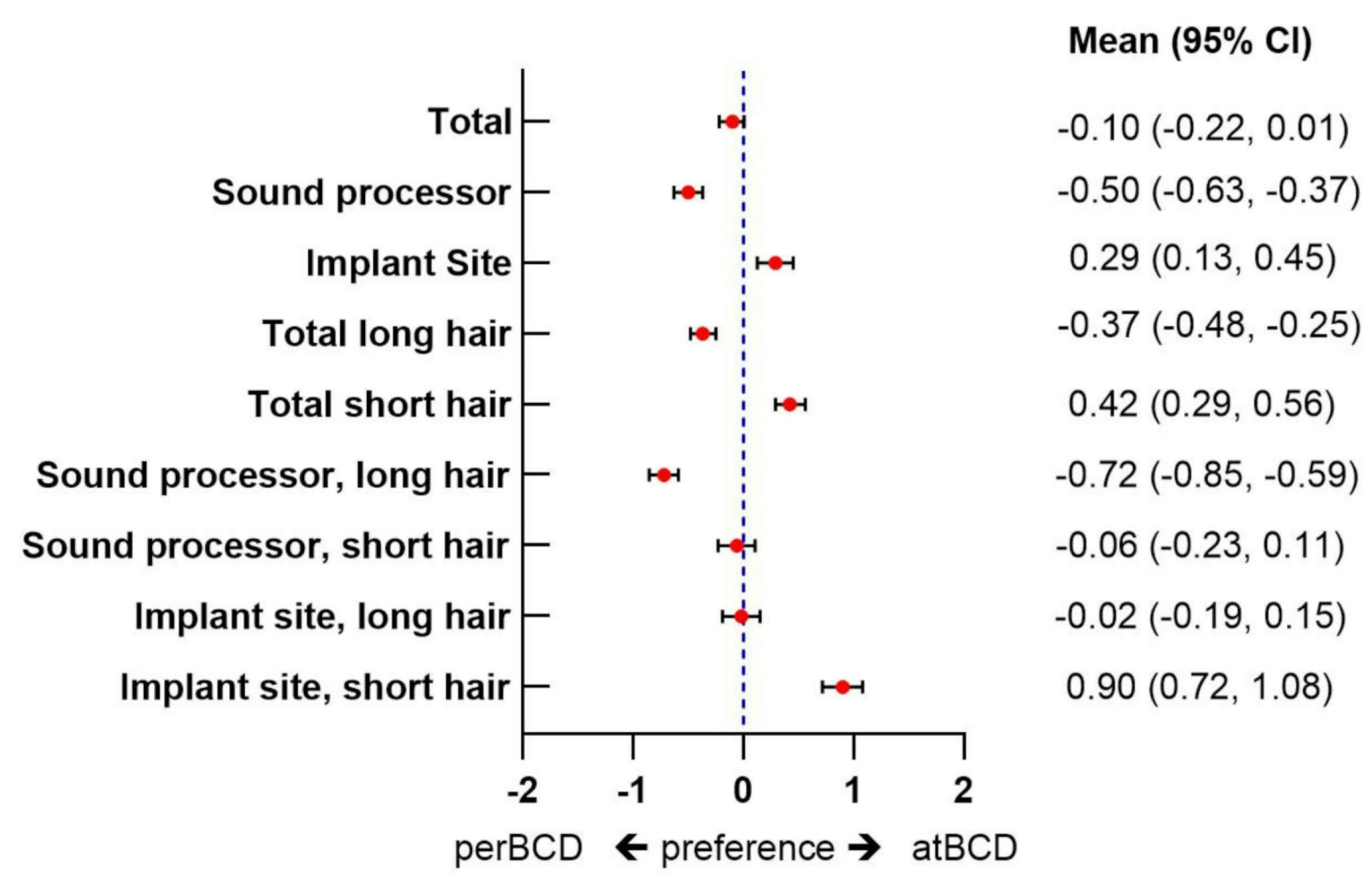
Mean preference scores for perBCD or atBCD in the total population. CI: confidence interval; perBCD: percutaneous bone conduction device; atBCD: active transcutaneous bone conduction device. Error bars represent 95% CI’s. A score of -2 reflects strong preference for perBCD and 2 a strong preference for atBCD. All mean scores that do not cross the 0 axis remain significantly different from 0 after Bonferroni correction

A preference for perBCD sound processors was observed (Fig. 2) (mean score − 0.50 (95% CI: -0.63, -0.37)). For the implant sites, atBCDs were preferred (mean score 0.29 (95% CI: 0.13, 0.45)). PerBCD sound processors were preferred in individuals with long hair (-0.72 (95% CI: -0.85, -0.59)). For pair wise comparisons showing the implant sites of individuals with short hair, atBCD was preferred (0.90 (95% CI: 0.72–1.08).

Cronbach’s alpha was 0.83 for the total score, 0.81 for the sub score showing sound processors and 0.88 for the sub score showing implant sites. Deletion of items did not lead to a substantial improvement of Cronbach’s alpha.

BCD users have a slight preference for perBCDs whilst non-users do not (mean score − 0.25 (-0.44, -0.09) versus 0.03 (-0.11, 0.17), respectively, *p* = 0.02) (Table 2). For implant sites, BCD-users have no preference (mean score − 0.03 (95% CI: -0.27, 0.21) whilst non-users prefer atBCDs (mean score 0.60 (95% CI: 0.20, 0.80). The effect size for the difference in preference for implant sites between BCD users and non-users is moderate (cohen’s d -0.56). BCD-users had a weaker preference for atBCD implant sites of short haired individuals when compared to non-users (0.42 (95% CI: 0.15, 0.69) versus 1.37 (95% CI: 1.17, 1.57), cohen’s d -0.77).

**Table 2:** Differences between BCD users and non-users in mean preference scores for perBCD or atBCD

Property	Mean score BCD users ^a^	Mean score non-users ^a^	*P*-value ^b^	Cohen’s d
Total	-0.25 (-0.44, -0.09)	0.03 (-0.11, 0.17)	0.02	-0.34
Sound processor	-0.46 (-0.67, -0.26)	-0.54 (-0.70, -0.37)	0.72	0.08
Implant Site	-0.03 (-0.27, 0.21)	0.60 (0.40, 0.80)	< 0.01*	-0.56
Total long hair	-0.48 (-0.66, -0.31)	-0.26 (-0.41, -0.11)	0.48	-0.27
Total short hair	0.23 (0.02, 0.45)	0.60 (0.44. 0.80)	< 0.01*	-0.38
Sound processor, long hair	-0.71 (-0.92, -0.51)	-0.73 (-0.88, -0.56)	0.60	0.01
Sound processor, short hair	0.04 (-0.23, 0.31)	-0.16 (-0.38, 0.06)	0.06	-0.38
Implant site, long hair	-0.25 (-0.50, 0.00)	0.21 (-0.01, 0.45)	0.22	0.16
Implant site, short hair	0.42 (0.15, 0.69)	1.37 (1.17, 1.57)	< 0.01*	-0.77

BCD: bone conduction device; ^a^ Mean scores on a scale of -2 to 2 were presented with mean and 95% confidence intervals; ^b^*p*-values were derived from two-sample t-tests or Mann-Whitney U in case of skewed data and substantial differences between parametric and non-parametric tests; asterisk denotes *p* values that are statistically significant after Bonferroni correction

### Valued importance of BCD appearance

**Fig. 3:**
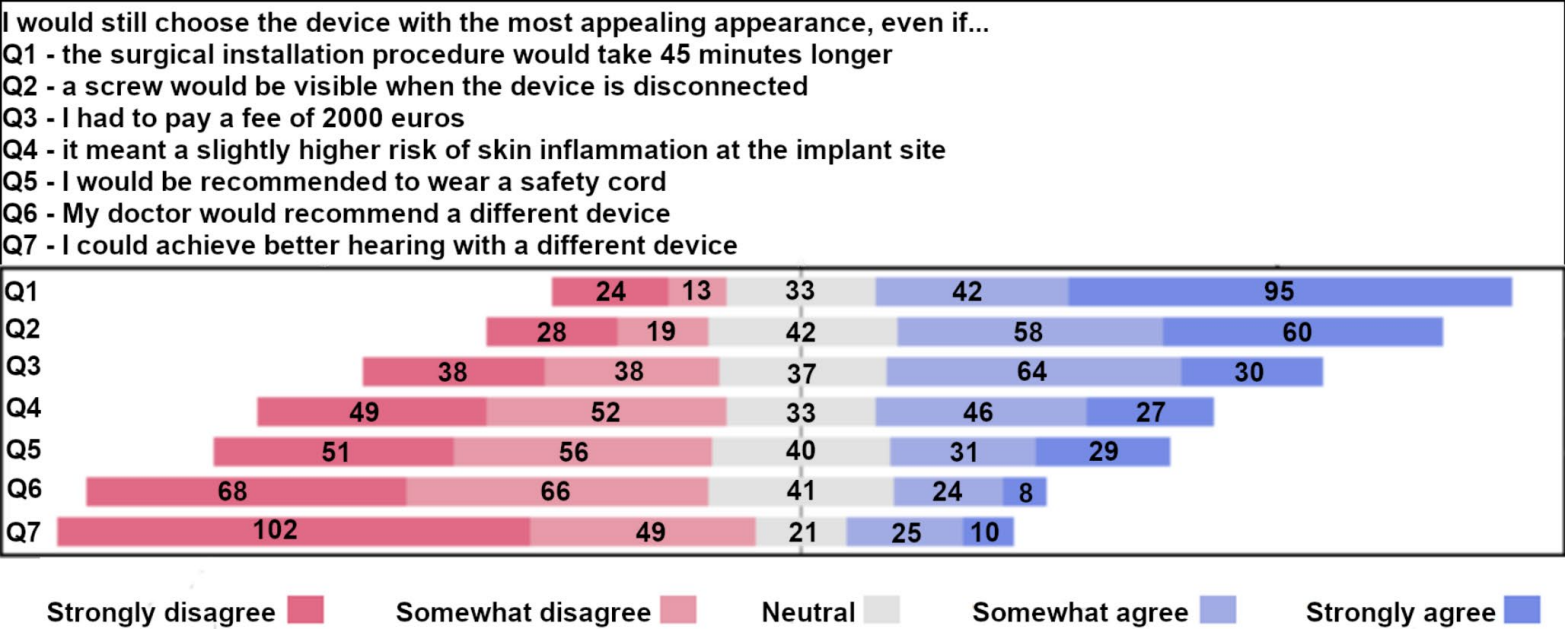
Responses of the total population on questions about the importance of BCD appearance. Q: question. In the bar chart, counts are presented, with the total count in every question amounting to *n* = 207

Most participants agreed with the statements ‘I would still choose the device with the most appealing appearance, even if the surgical installation procedure takes 45 minutes longer’ (*n* = 137, 66.2%) (Fig. 3). Conversely, most participants disagreed with the statement ‘I would still choose the device with the most appealing appearance, even if I could achieve better hearing with a different device’ ( *n* = 151, 73.0%).

Cronbach’s alpha for the importance of BCD appearance items was 0.65. Deletion of items did not lead to a substantially higher reliability measured by Cronbach’s alpha. All items had low to moderate correlation with the total score (Supplement Material B1, Correlation of item scores to total scores for participant valued BCD appearance).

Participant age was negatively correlated with the total mean score for valued BCD appearance (Pearson’s r -0.19, 95% CI -0.31 to -0.05) (Supplementary Material B2, Participant characteristics and total score of participant valued BCD appearance). Furthermore, BCD users with an outer ear malformation had a higher mean when compared to those without (mean difference 0.58 (95% CI 0.06 to 1.11, *P* = 0.03). However, these dependencies did not hold after Bonferroni correction.

## Discussion

Both Bone Conduction Device (BCD) users and non-users preferred the appearance of percutaneous BCD (perBCD) sound processors. Non-users preferred the implant sites of active transcutaneous BCDs (atBCDs). BCD-users had an overall preference for perBCDs whilst non-users did not. Most participants found the appearance of a BCD less important than optimal hearing or the advice of their counsellor. The appearance of a BCD may be more important for younger individuals or those affected by an outer ear malformation.

### Appearance preferences

The stronger preference for perBCDs by BCD-users in comparison to non-users may be explained by the occurrence of a confirmation bias. Almost all BCD-users used a perBCD. It has been established that persons tend to answer in a way that is consistent with decisions made in the past [20]. As such, having made the investment associated with the uptake and use of a perBCD, one may be more likely to respond in favor of this type. However, presence of a confirmation bias may only be confirmed if the questionnaire were to be conducted in a larger atBCD population as well.

Many studies have claimed that transcutaneous systems have a preferable appearance [12, 14–16]. This claim is contradicted by the total preference and sound processor-based preference for perBCDs observed in this study. Contrastingly, perBCD implant site appearance does seem inferior to transcutaneous implant sites, which is line with previous work [13].

The difference in perBCD versus atBCD preference based on hair style of the depicted model may be explained by the following. Newer perBCDs have a smaller surface area than atBCDs but protrude more due to the abutment. Studies show that the least visible device is commonly preferred [23]. For individuals with long hair, the protrusion is less noticeable, making the smaller perBCD the preferred device. Contrarily, for those with short or no hair, the protrusion is more visible, making atBCDs the preferred option in the current evaluation.

### Importance of appearance

Most participants found optimal hearing rehabilitation more important than having a BCD with the most appealing appearance. It has been described that concerns regarding self-image and perception by others due to the use of a BCD are outweighed by increased social functioning gained by hearing rehabilitation [29]. If this benefit is not perceived, disadvantages such as the appearance of a device may logically bear greater weight for the user.

Participants also valued the advice of their doctor during the decision-making process, highlighting the need for unbiased counselling when discussing perBCD and atBCD options. Fewer than one fourth of participants would not want the BCD with the most appealing appearance if a screw were visible when the device is disconnected. In previous work, authors do mention the visibility of an abutment to deter patients [12–13]. For these individuals, a atBCD would be a valuable alternative to a perBCD.

Whether individuals with younger age or an outer ear malformation attribute greater value to the appearance of a BCD could not be confirmed with certainty in this study. However, such associations may exist. To illustrate, studies have linked younger age with greater stigmatization in the context of conventional hearing aids [24]. Further, younger persons attribute greater value to appearance than their older counterparts in general [30]. Persons with an outer ear malformation experience social difficulty and psychological problems due to appearance issues [31–32]. Having faced the negative consequences of an atypical appearance, these individuals may place greater emphasis on wanting to look ‘normal’.

### Strengths and limitations

A strength was the recruitment of both BCD-users and non-users, which provided a relevant nuance to our findings. Another strength was the primary construction of the questionnaire in English. By doing so, we provide a solid basis for future studies on this subject.

There are several limitations to this study and areas for improvement concerning the questionnaire. First, real-life patients were used as models for pairwise comparisons of perBCD versus atBCD. As a result, the comparisons lacked absolute objectivity, displaying differences such as age, earrings, or slight skin inflammation at the implant site. On the other hand, one might find that this rightly depicts reality.

Another limitation regarding the pair-wise comparisons was the absence of a short-haired model using an Osia 2 system. Due to this, the finding that atBCD implant sites were preferred in short haired individuals should be interpreted with care. The Osia 2 system shows more bulk beneath the skin (i.e.,lies more proud due to different implantation techniques) than the Sentio system, tending to increase its visibility, although this is somewhat dependent on the patients’ skull. Assuming that the most preferred implant site is the one with least visibility, inclusion of a short haired individual with an Osia may have decreased the observed atBCD implant site preference.

A third limitation of this study relates to the question phrasing. Phrasing such as “slightly higher risk of skin inflammation” or “better hearing” might have been interpreted differently by each participant, potentially reducing inter-responder reliability. In future studies, it may be relevant to avoid such questions, or rephrase them in a manner that facilitates more uniform interpretation.

### Future implications

At present, manufacturers have shifted their focus from percutaneous to active transcutaneous BCDs. Numerous studies examine the objective differences between these device types, but personal preferences remain relatively underexplored. This study suggests that common assumptions, such as the superior appearance of atBCDs compared to perBCDs, may not fully reflect reality. Gaining insight into patients’ personal preferences when choosing between device types is essential for enhancing the decision-making process.

To gain a better understanding of these preferences, future studies should address several aspects. First, appearance preferences should be investigated among atBCD users, as they were underrepresented in this study. Second, since this study featured a local population of BCD users and non-users, it would be valuable to explore appearance preferences in populations with different cultural or socioeconomic backgrounds. Third, as appearance is only one factor, studies should also explore other personal preferences, such as financial concerns. Combining these research efforts could ultimately aid in developing a decision-making tool for choosing between perBCDs and atBCDs.

## Conclusion

This newly designed questionnaire suggests that the appearance of perBCD sound processors is preferred by both BCD-users and non-users. Non-users preferred the implant site of atBCDs. The appearance of a BCD is considered less important than optimal hearing or the advice of their counsellor. The appearance of a BCD may be more important for younger individuals or those affected by an outer ear malformation. Further studies, involving a larger group of atBCD users and persons from other cultural or socioeconomic backgrounds, are necessary to confirm or contradict these findings.

## Electronic supplementary material

Below is the link to the electronic supplementary material.

Supplementary Material 1

Supplementary Material 2

## References

[1] Faber CADHT, de Wolf MJ, Hol CWCMK (2011) An overview of different systems: the bone-anchored hearing aid. *Implantable Bone Conduction Hearing Aids,* 71:22–31. 10.1159/000323577 21389701

[2] Reinfeldt S, Håkansson B, Taghavi H, Eeg-Olofsson M (2015) New developments in bone-conduction hearing implants: A review, Jan. 16, *Dove Medical Press Ltd*. 10.2147/MDER.S39691 10.2147/MDER.S39691 PMC4303401 25653565

[3] Hol MKS, Nelissen RC, Agterberg MJH, Cremers CWRJ, Snik AFM (2013) Comparison Between a New Implantable Transcutaneous Bone Conductor and Percutaneous Bone-Conduction Hearing Implant, *Otology & Neurotology*, vol. 34, no. 6, pp. 1071–1075, Aug. 10.1097/MAO.0b013e3182868608 10.1097/MAO.0b013e3182868608 23598702

[4] Cooper T, McDonald B, Ho A (2017) Passive Transcutaneous Bone Conduction Hearing Implants: A Systematic Review, Oct. 01, *Lippincott Williams and Wilkins*. 10.1097/MAO.0000000000001518 10.1097/MAO.0000000000001518 28719403

[5] Tjellström A, Lindström J, Hallén O, Albrektsson T, Brånemark PI (1981) Osseointegrated titanium implants in the temporal bone. A clinical study on bone-anchored hearing aids., *Am J Otol*, vol. 2, no. 4, pp. 304–10, Apr 6894824

[6] Sprinzl G et al (Aug. 2013) First European multicenter results with a new transcutaneous bone conduction hearing implant system. Otology Neurotology 34(6):1076–1083. 10.1097/MAO.0b013e31828bb541 10.1097/MAO.0b013e31828bb541 23714710

[7] Ellsperman SE, Nairn EM, Stucken EZ (May 2021) Review of bone conduction hearing devices. Audiol Res 11(2):207–219. 10.3390/audiolres11020019 10.3390/audiolres11020019 PMC8161441 34069846

[8] Amin N, Soulby AJ, Borsetto D, Pai I (2021) Longitudinal economic analysis of Bonebridge 601 versus percutaneous bone-anchored hearing devices over a 5-year follow-up period, *Clinical Otolaryngology*, vol. 46, no. 1, pp. 263–272, Jan. 10.1111/coa.13659 10.1111/coa.13659 33068331

[9] Nassiri AM et al (2024) Magnetic Resonance Imaging Artifact Associated With Transcutaneous Bone Conduction Implants: Cholesteatoma and Vestibular Schwannoma Surveillance, *Otolaryngology–Head and Neck Surgery*, vol. 170, no. 1, pp. 187–194, Jan. 10.1002/ohn.474 10.1002/ohn.474 37582349

[10] Magele A, Schoerg P, Stanek B, Gradl B, Sprinzl GM (2019) Active transcutaneous bone conduction hearing implants: systematic review and meta-analysis. Sep 01 2019 Public Libr Sci. 10.1371/journal.pone.0221484 10.1371/journal.pone.0221484 PMC6746395 31525208

[11] Taghavi H, Håkansson B, Reinfeldt S, Eeg-Olofsson M, Akhshijan S (Apr. 2012) Feedback analysis in percutaneous Bone-Conduction device and Bone-Conduction implant on a dry cranium. Otology Neurotology 33(3):413–420. 10.1097/MAO.0b013e3182487fc8 10.1097/MAO.0b013e3182487fc8 22410731

[12] Sprinzl G et al (2021) Long-Term, Multicenter Results With the First Transcutaneous Bone Conduction Implant, *Otology and Neurotology*, vol. 42, no. 6, pp. 858–866, Jul. 10.1097/MAO.0000000000003159 10.1097/MAO.0000000000003159 33989254

[13] Siau D, Dhillon B, Andrews R, Green KMJ (2015) Bone-anchored hearing aids and unilateral sensorineural hearing loss: Why do patients reject them? *Journal of Laryngology and Otology*, vol. 129, no. 4, pp. 321–325, Apr. 10.1017/S0022215115000602 10.1017/S0022215115000602 25776860

[14] Kruyt IJ (2020) Bone Conduction Devices-Reviewing the past, evaluating the present, considerations for the future

[15] Leterme G et al (2015) Contralateral routing of signal hearing aid versus transcutaneous bone conduction in single-sided deafness, *Audiology and Neurotology*, vol. 20, no. 4, pp. 251–260, Aug. 10.1159/000381329 10.1159/000381329 26021779

[16] Svagan M, Brzan PP, Rebol J (2019) Comparison of Satisfaction Between Patients Using Percutaneous and Transcutaneous Bone Conduction Devices, *Otology & Neurotology*, vol. 40, no. 5, pp. 651–657, Jun. 10.1097/MAO.0000000000002203 10.1097/MAO.0000000000002203 31083093

[17] Rasmussen J, Olsen SØ, Nielsen LH (2012) Evaluation of long-term patient satisfaction and experience with the Baha ^®^ bone conduction implant, *Int J Audiol*, vol. 51, no. 3, pp. 194–199, Mar. 10.3109/14992027.2011.635315 10.3109/14992027.2011.635315 22133063

[18] Saroul N, Gilain L, Montalban A, Giraudet F, Avan P, Mom T (2011) Patient satisfaction and functional results with the bone-anchored hearing aid (BAHA), *Eur Ann Otorhinolaryngol Head Neck Dis*, vol. 128, no. 3, pp. 107–113, Jun. 10.1016/j.anorl.2010.09.009 10.1016/j.anorl.2010.09.009 21601551

[19] Garcier M, Lavedrine A, Gagneux C, Eluecque T, Bozorg Grayeli A (2021) Bone-Anchored and Closed Skin Bonebridge Implant in Adults: Hearing Performances and Quality of Life, *Audiology and Neurotology*, vol. 26, no. 5, pp. 310–316, Sep. 10.1159/000512496 10.1159/000512496 33662952

[20] Jonas E, Schulz-Hardt S, Frey D, Thelen N (2001) Confirmation bias in sequential information search after preliminary decisions: an expansion of dissonance theoretical research on selective exposure to information. J Pers Soc Psychol 80(4):557–571. 10.103700022-3514.80.4.557 11316221 10.1037//0022-3514.80.4.557

[21] Zawawi F, Kabbach G, Lallemand M, Daniel SJ (Feb. 2014) Bone-anchored hearing aid: why do some patients refuse it? Int J Pediatr Otorhinolaryngol 78(2):232–234. 10.1016/j.ijporl.2013.11.010 10.1016/j.ijporl.2013.11.010 24377490

[22] Saroul N, Akkari M, Pavier Y, Gilain L, Mom T (2014) Baha-mediated rehabilitation of patients with unilateral deafness: selection criteria. Audiol Neurotology 19(2):85–90. 10.1159/000354272 10.1159/000354272 24401757

[23] Almufarrij I, Munro KJ, Dawes P, Stone MA, Dillon H (Jan. 2019) Direct-to-Consumer hearing devices: capabilities, costs, and cosmetics. Trends Hear 23:233121651985830. 10.1177/2331216519858301 10.1177/2331216519858301 PMC6614949 31280709

[24] Werner P (2016) Stigma regarding hearing loss and hearing aids: A scoping review., *Stigma Health*, vol. 1, no. 2, pp. 59–71, May 10.1037/sah0000022

[25] Elwyn G et al (Oct. 2012) Shared decision making: A model for clinical practice. J Gen Intern Med 27(10):1361–1367. 10.1007/s11606-012-2077-6 10.1007/s11606-012-2077-6 PMC3445676 22618581

[26] Beers E, Lee Nilsen M, Johnson JT (Aug. 2017) The role of patients. Otolaryngol Clin North Am 50(4):689–708. 10.1016/j.otc.2017.03.006 10.1016/j.otc.2017.03.006 28571664

[27] Joosten EAG, DeFuentes-Merillas L, de Weert GH, Sensky T, van der Staak CPF, de Jong CAJ (2008) Systematic review of the effects of shared Decision-Making on patient satisfaction, treatment adherence and health status. Psychother Psychosom 77(4):219–226. 10.1159/000126073 18418028 10.1159/000126073

[28] Beaton DE, Bombardier C, Guillemin F, Ferraz MB (1976) Guidelines for the Process of Cross-Cultural Adaptation of Self-Report Measures, *Spine (Phila Pa* vol. 25, no. 24, pp. 3186–3191, 2000 10.1097/00007632-200012150-00014 11124735

[29] Almugathwi M, Wearden A, Green K, Hill-Feltham P, Powell R (2020) Online support group users’ perceptions and experiences of bone-anchored hearing aids (BAHAs): a qualitative study, *Int J Audiol*, vol. 59, no. 11, pp. 850–858, Oct. 10.1080/14992027.2020.1771440 10.1080/14992027.2020.1771440 32522055

[30] Quittkat HL, Hartmann AS, Düsing R, Buhlmann U, Vocks S (2019) Body Dissatisfaction, Importance of Appearance, and Body Appreciation in Men and Women Over the Lifespan, *Front Psychiatry*, vol. 10, Dec. 10.3389/fpsyt.2019.00864 10.3389/fpsyt.2019.00864 PMC6928134 31920737

[31] Jiamei D, Jiake C, Hongxing Z, Wanhou G, Yan W, Gaifen L (Jan. 2008) An investigation of psychological profiles and risk factors in congenital microtia patients. J Plast Reconstr Aesthetic Surg 61:S37–S43. 10.1016/j.bjps.2007.09.002 10.1016/j.bjps.2007.09.002 17980688

[32] Johns AL, Lewin SL, Im DD (May 2017) Teasing in younger and older children with microtia before and after ear reconstruction. J Plast Surg Hand Surg 51(3):205–209. 10.1080/2000656X.2016.1222294 10.1080/2000656X.2016.1222294 27609237

