# Supplementary material for: Participant valued appearance of bone conduction devices: a comparison between percutaneous and transcutaneous systems
Source: Eur Arch Otorhinolaryngol. 2025 Mar 22;282(9):4467–75. doi: 10.1007/s00405-025-09335-7 (PMC12423119; doi:10.1007/s00405-025-09335-7)
Supplement: Supplementary file 1 — Supplementary Material 1 [file 405_2025_9335_MOESM1_ESM.pdf]

Supplementary material A) English version of the questionnaire

Domain 1

How old are you?

What is your gender?

- Male
- Female
- Other
- I prefer not to say

Would you say that your hair is long?

- Yes
- No

Have you ever heard of a 'bone conduction device' (BCD)?

- Yes
- No

Do you or have you ever used a bone conduction device?

- ○ Yes
- ○ No

Do you suffer from hearing loss that was confirmed by a hearing care facility or ENT department?

- ○ Yes
- ○ No

Do you use a hearing aid?

- ○ Yes
- ○ No

Which bone conduction device do you currently use?

- ○ Not applicable - I do not use a bone conduction device
- ○ Ponto (3,4,5) SP or Mini (Oticon Medical)
- ○ BAHA (4,5,6) or Max (Cochlear Limited)
- ○ Sentio (Oticon Medical)
- ○ OSIA (1,2) (Cochlear Limited)
- ○ BoneBridge (601,602) (MedEL)
- ○ BAHA Attract (Cochlear Limited)

- [ ] Sophono Alpha 2 (Medtronic)
- [ ]

How many hours per day do you use your bone conduction device?

- [ ] None
- [ ] Between 0-1 hour per day
- [ ] 1-4 hours per day
- [ ] 4-8 hours per day
- [ ] 8-16 hours per day

Has your outer ear developed differently due to a birth defect (such as microtia or congenital atresia)?

- [ ] Yes
- [ ] No
- [ ] I do not know

Are you deaf in one ear?

- [ ] Yes
- [ ] No

Is the hearing loss in one ear caused by a problem of your inner ear or auditory nerve?

- ○ Yes
- ○ No
- ○ I do not know

Domain 2

In the following section, pictures of bone conduction devices are shown. Bone conduction devices are a kind of hearing aid.
You will see comparisons showing one type of bone conduction device on the left (labeled with the letter A) and one type on the right (labeled with the letter B). You will be asked to indicate whether you prefer the appearance of the device on the left (A) or the right side (B).

Do you prefer the appearance of the device on the left (A) or on the right (B)?

Try to base your preference only on the actual devices, ignoring any differences in the persons shown.

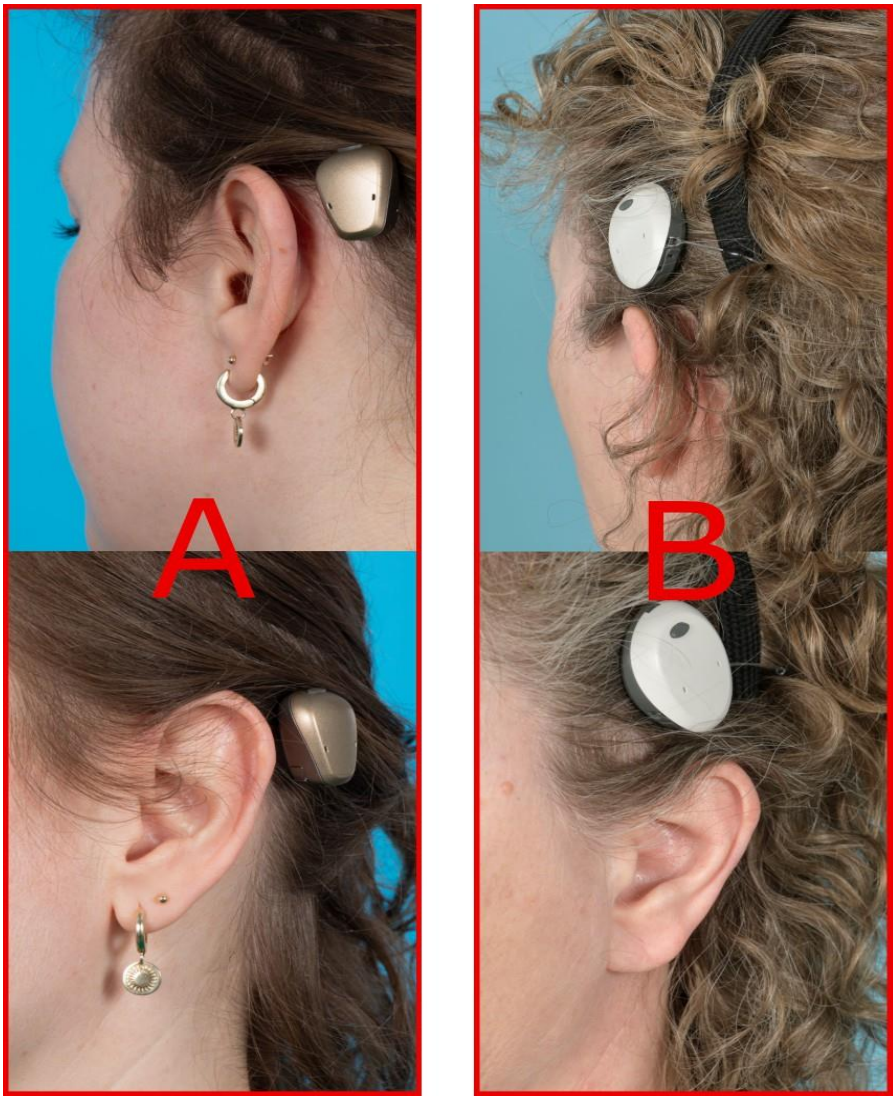

Much prefer A	Slightly prefer A	No preference	Slightly prefer B	Much prefer B
○	○	○	○	○

Do you prefer the appearance of the device on the left (A) or on the right (B)?

Try to base your preference only on the actual devices, ignoring any differences in the persons shown.

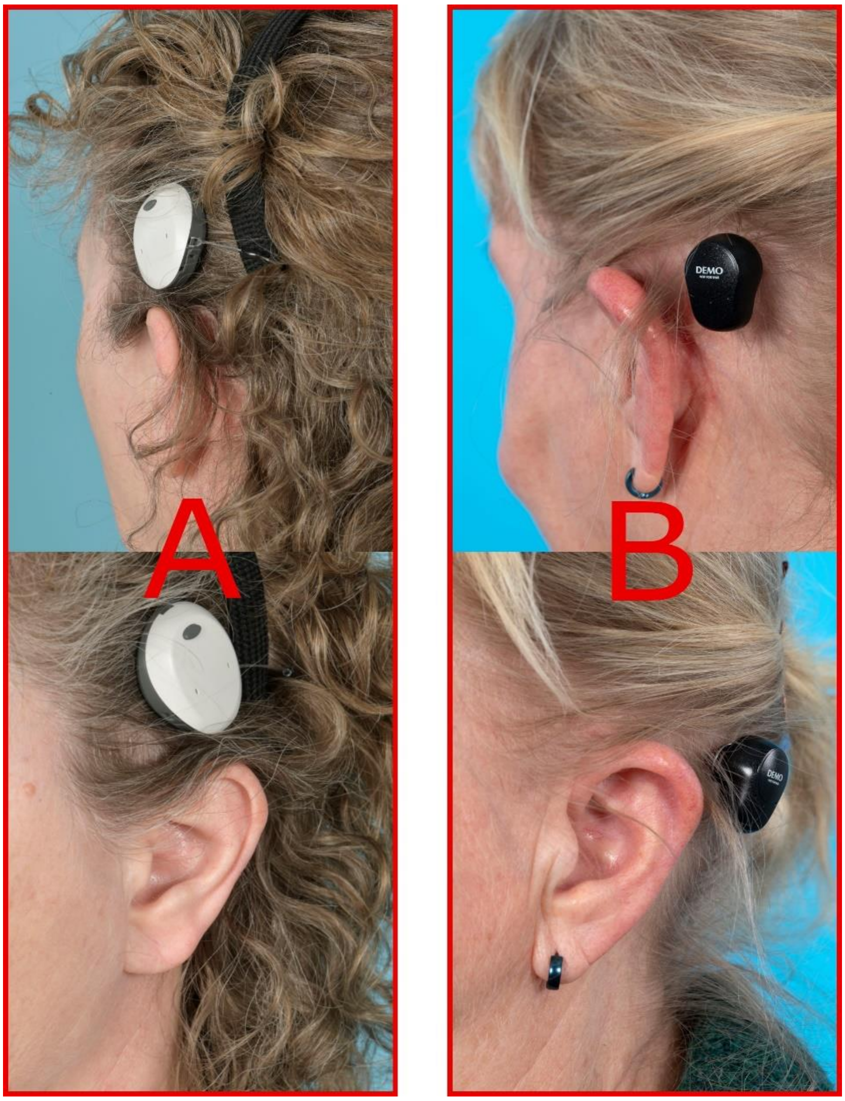

Much prefer A	Slightly prefer A	No preference	Slightly prefer B	Much prefer B
○	○	○	○	○

Do you prefer the appearance of the device on the left (A) or on the right (B)?

Try to base your preference only on the actual devices, ignoring any differences in the persons shown.

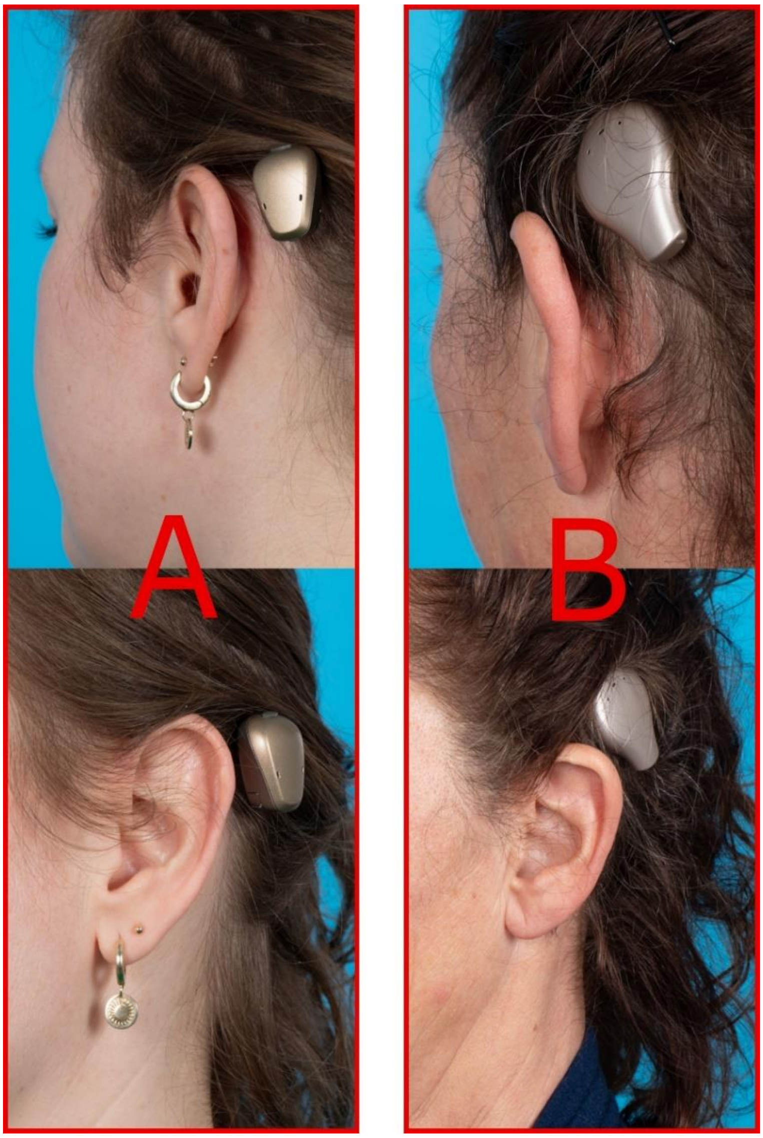

Much prefer A	Slightly prefer A	No preference	Slightly prefer B	Much prefer B
○	○	○	○	○

Do you prefer the appearance of the device on the left (A) or on the right (B)?

Try to base your preference only on the actual devices, ignoring any differences in the persons shown.

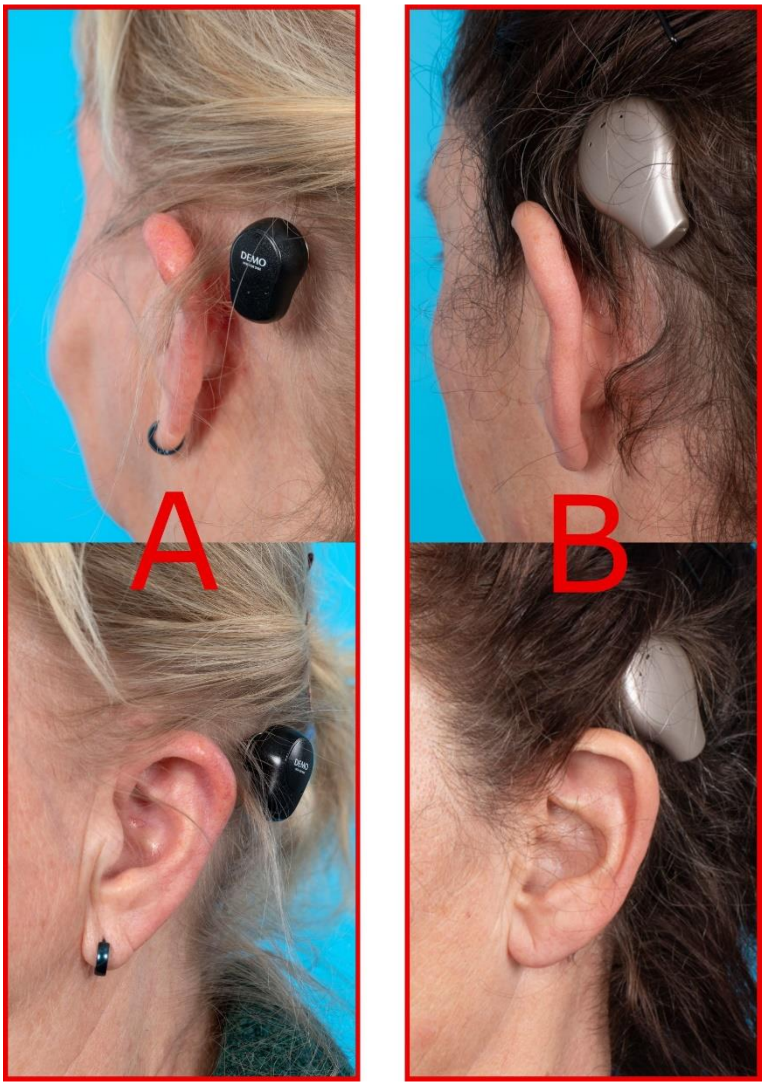

Much prefer A	Slightly prefer A	No preference	Slightly prefer B	Much prefer B
○	○	○	○	○

Do you prefer the appearance of the device on the left (A) or on the right (B)?

Try to base your preference only on the actual devices, ignoring any differences in the persons shown.

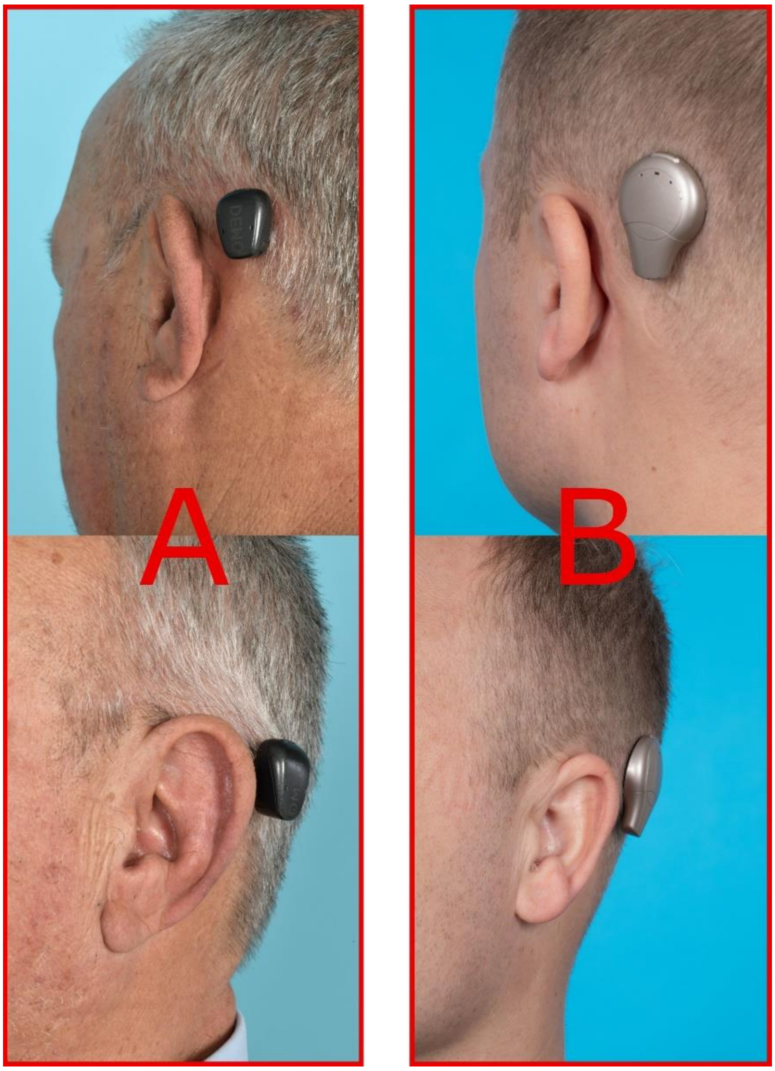

Much prefer A	Slightly prefer A	No preference	Slightly prefer B	Much prefer B
○	○	○	○	○

Do you prefer the appearance of the device on the left (A) or on the right (B)?

Try to base your preference only on the actual devices, ignoring any differences in the persons shown.

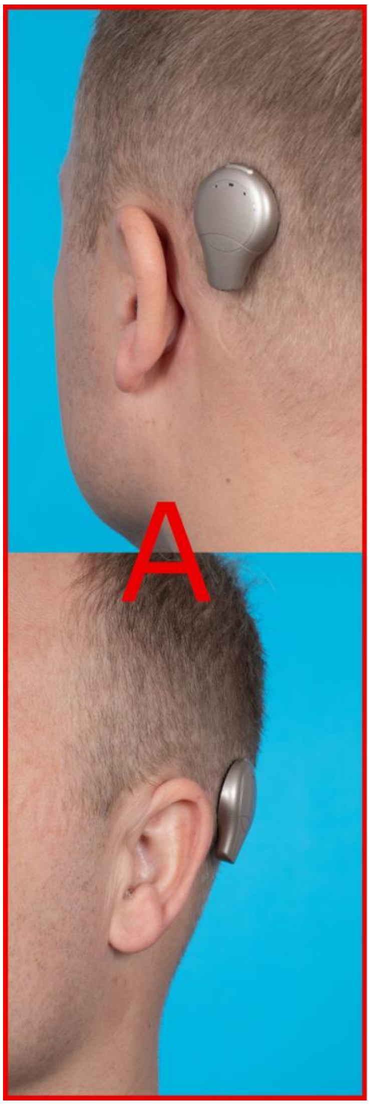

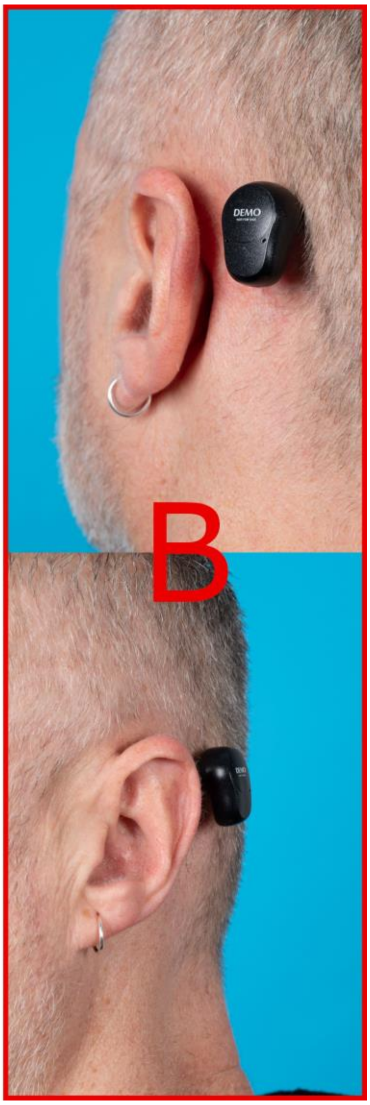

Much prefer A	Slightly prefer A	No preference	Slightly prefer B	Much prefer B
○	○	○	○	○

In the following pictures, the sound processor is detached. Behind the ear, you may notice the implant site.

Do you prefer the appearance of the implant site behind the ear on the left (A) picture or on the right (B)?

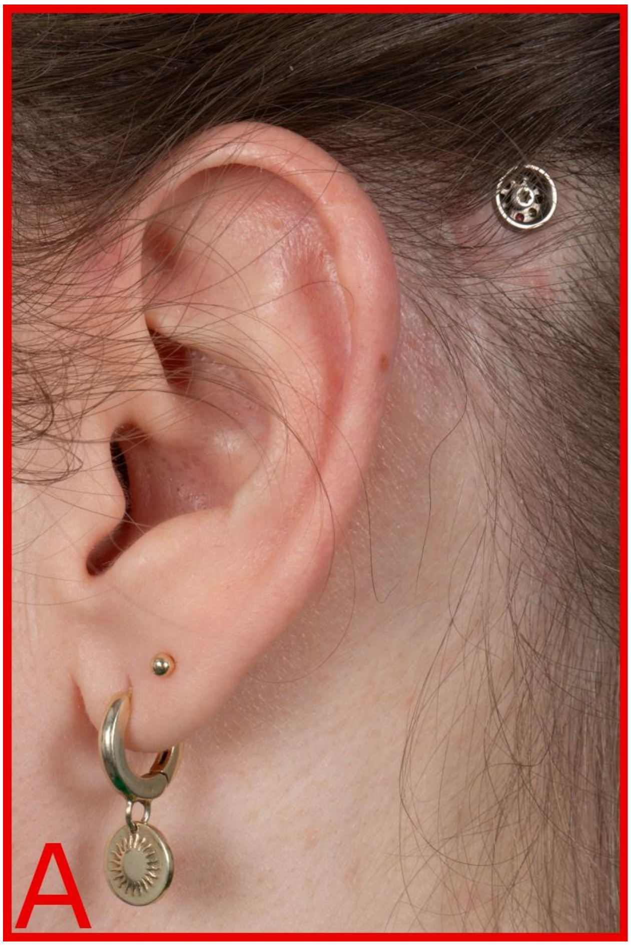

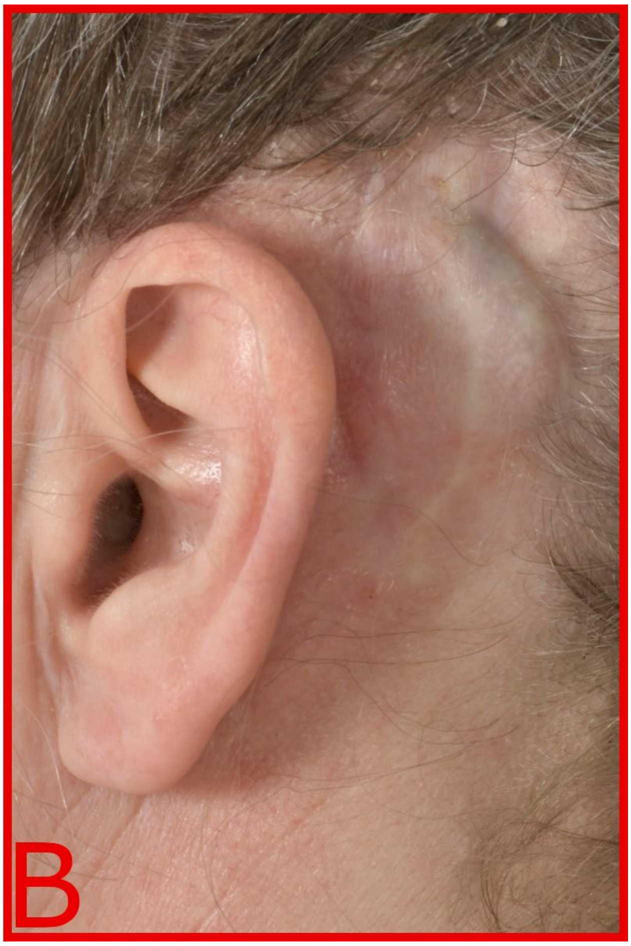

Much prefer A	Slightly prefer A	No preference	Slightly prefer B	Much prefer B
○	○	○	○	○

In the following pictures, the sound processor is detached. Behind the ear, you may notice the implant site.

Do you prefer the appearance of the implant site behind the ear on the left (A) picture or on the right (B)?

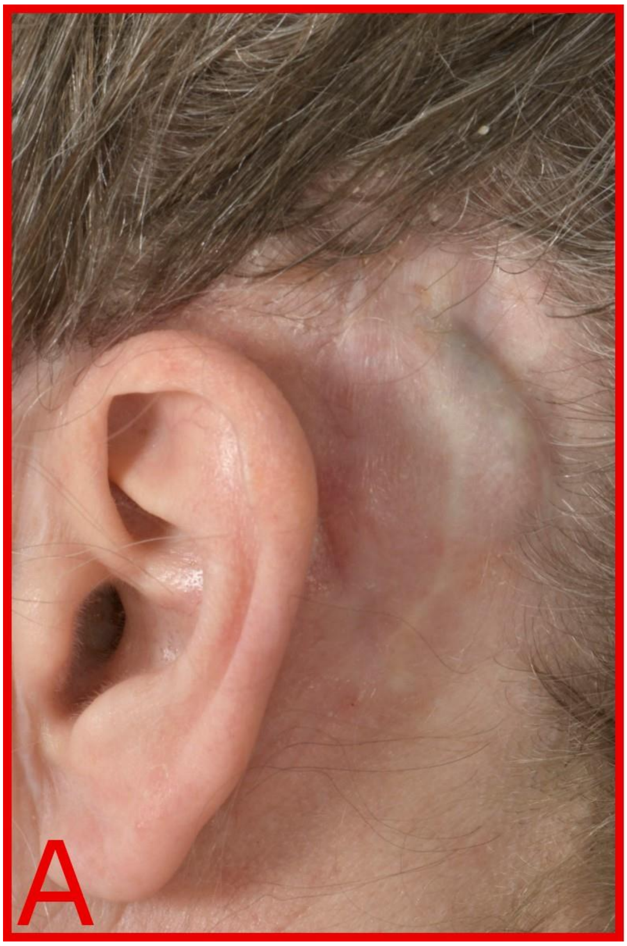

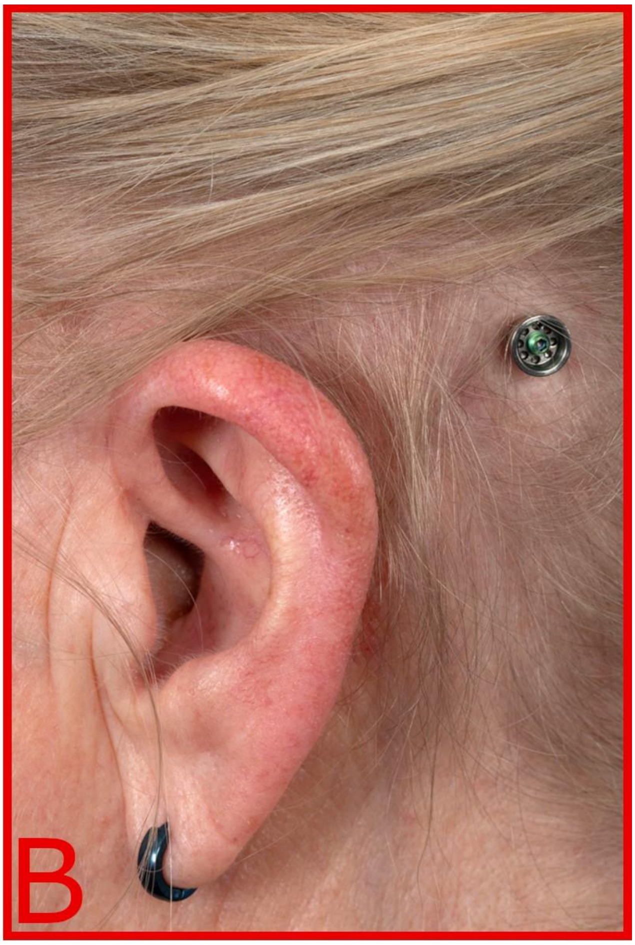

Much prefer A	Slightly prefer A	No preference	Slightly prefer B	Much prefer B
○	○	○	○	○

In the following pictures, the sound processor is detached. Behind the ear, you may notice the implant site.

Do you prefer the appearance of the implant site behind the ear on the left (A) picture or on the right (B)?

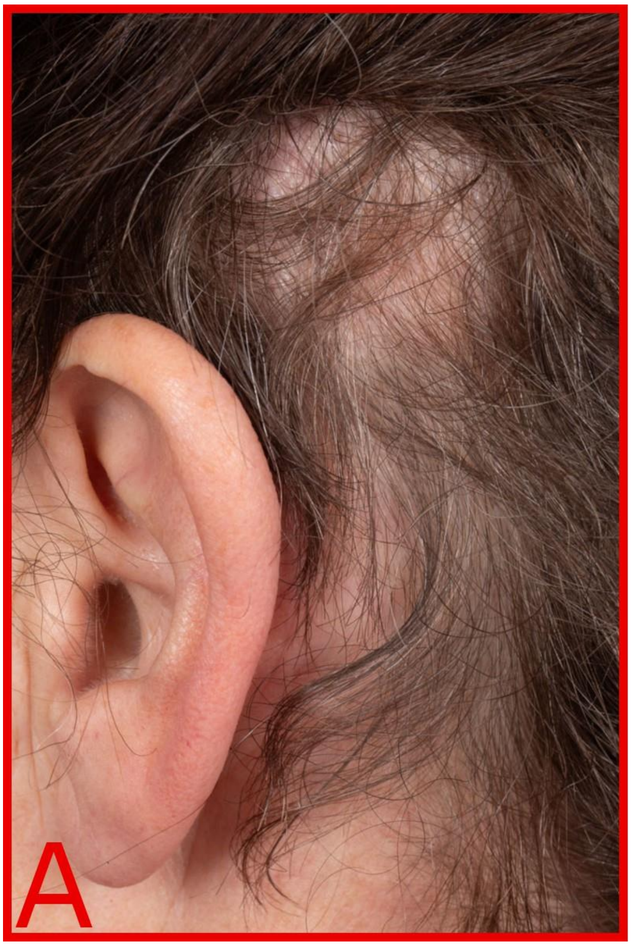

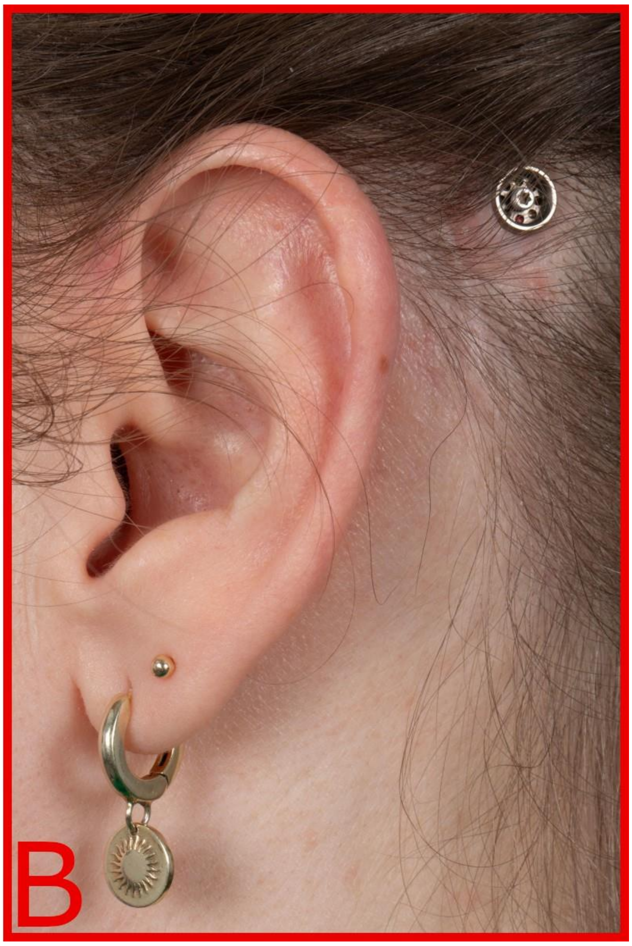

Much prefer A	Slightly prefer A	No preference	Slightly prefer B	Much prefer B
○	○	○	○	○

In the following pictures, the sound processor is detached. Behind the ear, you may notice the implant site.

Do you prefer the appearance of the implant site behind the ear on the left (A) picture or on the right (B)?

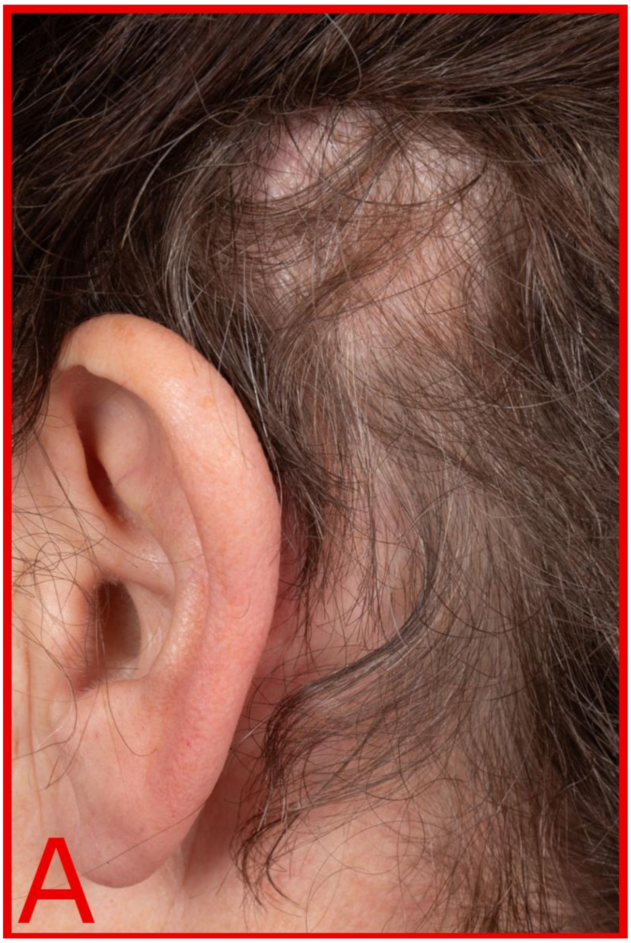

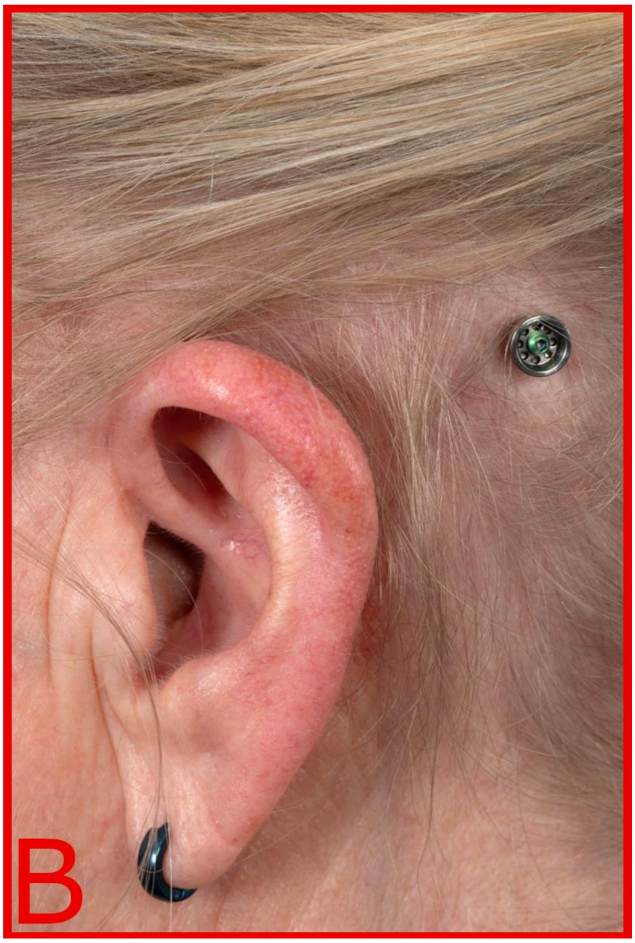

Much prefer A	Slightly prefer A	No preference	Slightly prefer B	Much prefer B
○	○	○	○	○

In the following pictures, the sound processor is detached. Behind the ear, you may notice the implant site.

Do you prefer the appearance of the implant site behind the ear on the left (A) picture or on the right (B)?

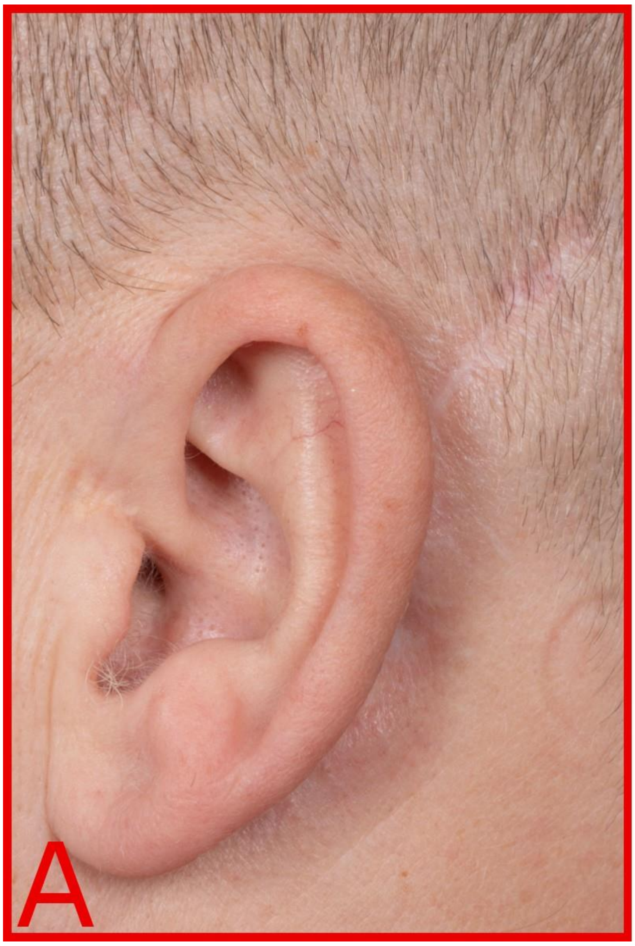

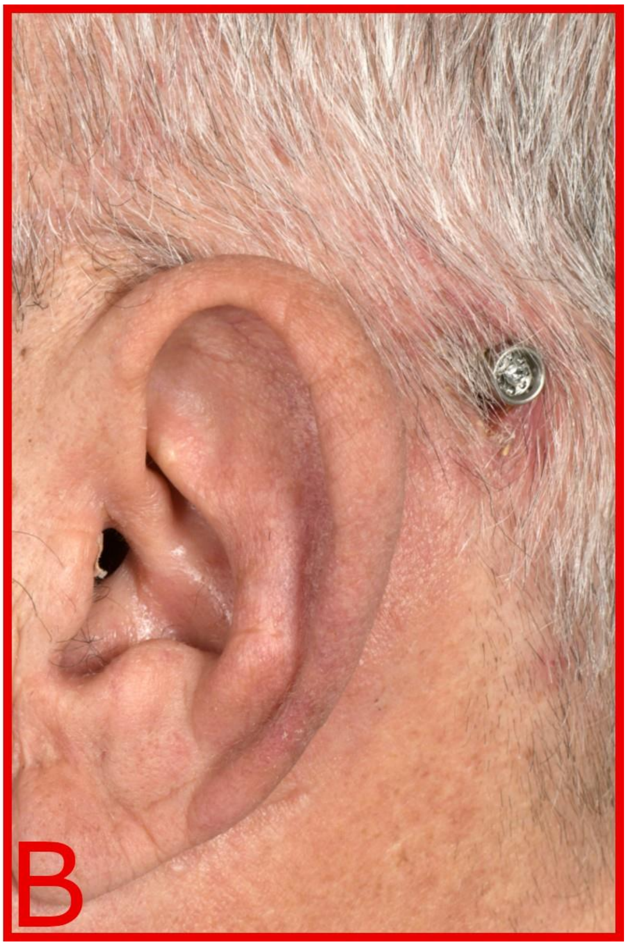

Much prefer A	Slightly prefer A	No preference	Slightly prefer B	Much prefer B
○	○	○	○	○

In the following pictures, the sound processor is detached. Behind the ear, you may notice the implant site.

Do you prefer the appearance of the implant site behind the ear on the left (A) picture or on the right (B)?

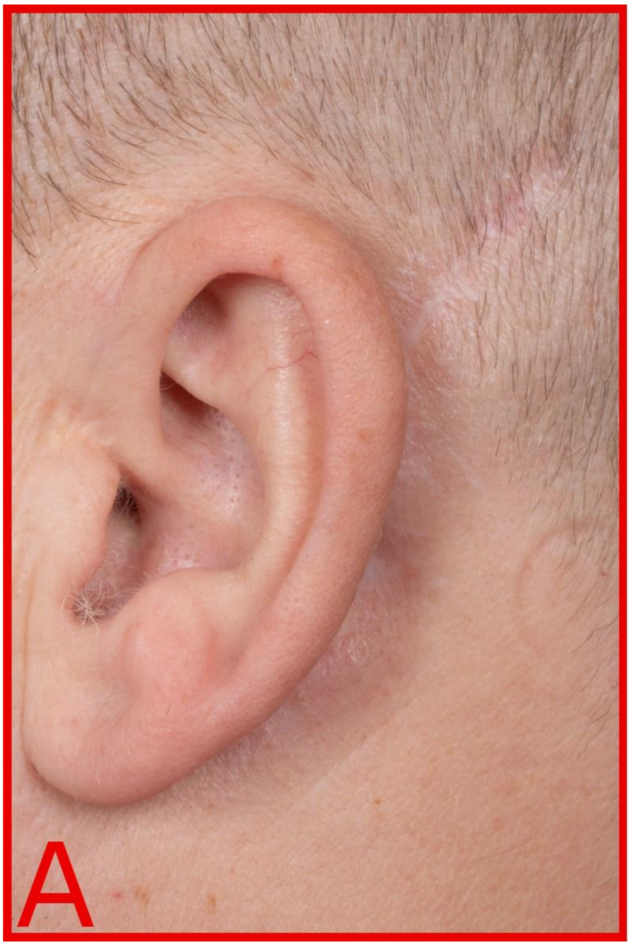

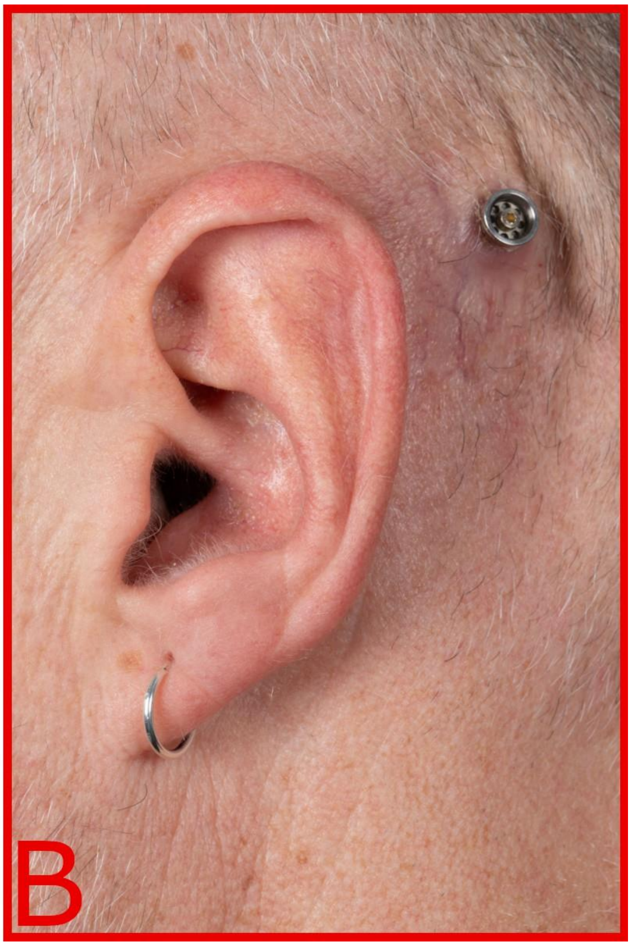

Much prefer A	Slightly prefer A	No preference	Slightly prefer B	Much prefer B
○	○	○	○	○

Domain 3

Both types of bone conduction devices have advantages and disadvantages. As a patient, you must choose one of these two types. Imagine that you are a patient and that you have to make this choice. Take another look at the bone conduction devices shown below and determine which type has the most appealing appearance. Then, respond to the folowing statements.

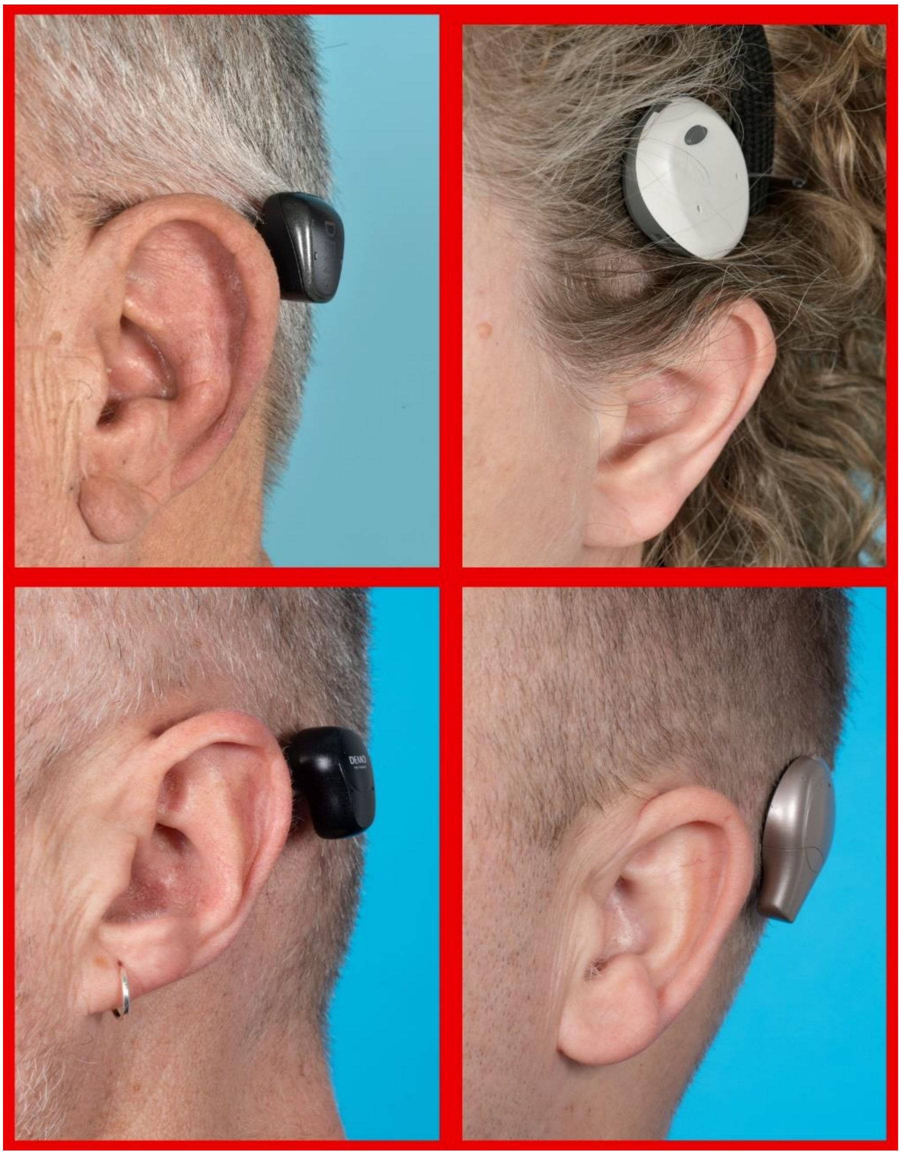

I would still choose the device with the most appealing appearance, even if I had to pay a fee of 2000 euros.

.	Strongly disagree	Somewhat disagree	Neither agree nor disagree	Somewhat agree	Strongly agree
	○	○	○	○	○

I would still choose the device with the most appealing appearance, even if I could achieve better hearing with a different device.

.	Strongly disagree	Somewhat disagree	Neither agree nor disagree	Somewhat agree	Strongly agree
	○	○	○	○	○

I would still choose the device with the most appealing appearance, even if my doctor would recommend a different device.

.	Strongly disagree	Somewhat disagree	Neither agree nor disagree	Somewhat agree	Strongly agree
	○	○	○	○	○

I would still choose the device with the most appealing appearance, even if the surgical installation procedure takes 45 minutes longer.

.	Strongly disagree	Somewhat disagree	Neither agree nor disagree	Somewhat agree	Strongly agree
	○	○	○	○	○

I would still choose the device with the most appealing appearance, even if it meant a slightly higher risk of skin inflammation at the implant site.

.	Strongly disagree	Somewhat disagree	Neither agree nor disagree	Somewhat agree	Strongly agree
	○	○	○	○	○

I would still choose the device with the most appealing appearance, even if a screw would be visible when the device is not connected (as seen below).

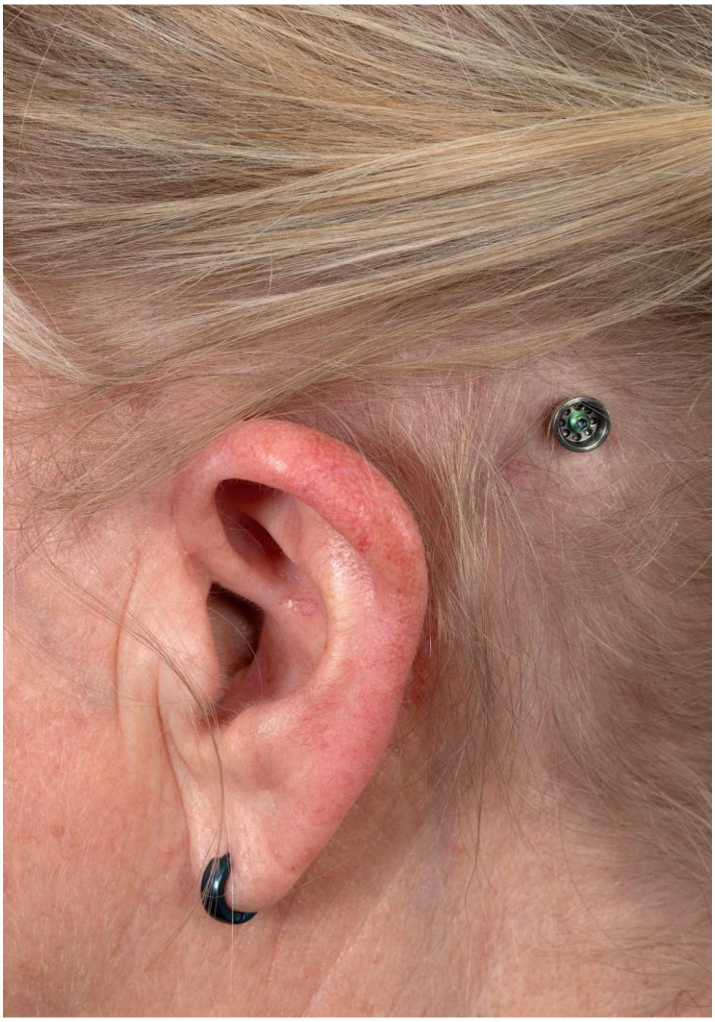

.	Strongly disagree	Somewhat disagree	Neither agree nor disagree	Somewhat agree	Strongly agree
	○	○	○	○	○

I would still choose the device with the most appealing appearance, even if I would be recommended to wear a safety cord (as seen below).

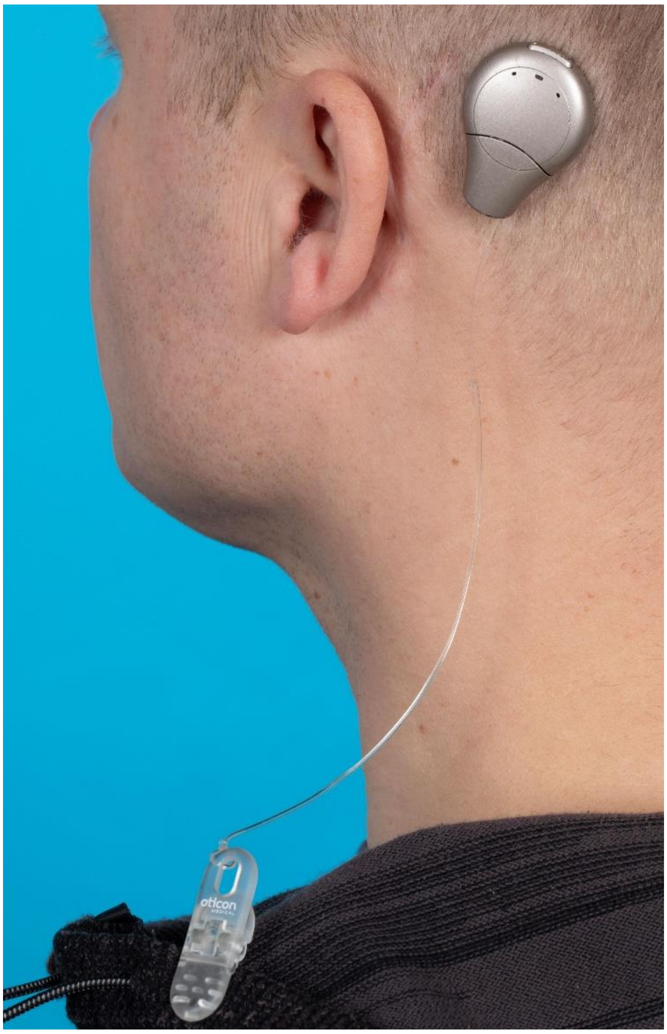

.	Strongly disagree	Somewhat disagree	Neither agree nor disagree	Somewhat agree	Strongly agree
	○	○	○	○	○

Domain 4

Do you have any comments on your considerations when deciding between the two types of bone conduction devices?

Do you have any remarks about this questionnaire?
