# Supplementary material for: Participant valued appearance of bone conduction devices: a comparison between percutaneous and transcutaneous systems
Source: Eur Arch Otorhinolaryngol. 2025 Mar 22;282(9):4467–75. doi: 10.1007/s00405-025-09335-7 (PMC12423119; doi:10.1007/s00405-025-09335-7)
Supplement: Supplementary file 2 — Supplementary Material 2 [file 405_2025_9335_MOESM2_ESM.pdf]

Supplementary Material B1) Correlation of item scores to total scores for participant valued BCD appearance.

**I would still choose the device with the most appealing appearance, even if…**	**Item-Total Correlation** [a]	**p-value** [b]
I had to pay a fee of 2000 euros.	0.53 (0.45, 0.64)	< 0.01
I could achieve better hearing with a different device.	0.58 (0.48, 0.66)	< 0.01
my doctor would recommend a different device.	0.65 (0.56, 0.72)	< 0.01
the surgical installation procedure takes 45 minutes longer.	0.62 (0.53, 0.70)	< 0.01
it meant a slightly higher risk of skin inflammation at the implant site.	0.65 (0.57, 0.73)	< 0.01
a screw would be visible when the device is not connected.	0.56 (0.46, 0.65)	< 0.01
I would be recommended to wear a safety cord.	0.39 (0.27, 0.50)	< 0.01

[a] Pearsons r correlation coefficient of item score to total score was presented with 95% Confidence Intervals. [b] p-values were derived from the H0: Pearson's r = 0.

Supplementary Material B2) Participant characteristics and total score of participant valued BCD appearance.

**Characteristic**	**Statistical test**	**Total sum score** [a]	**P value** [b]
Age	Pearson's r	-0.18 (-0.31, -0.05)	0.01
Sex (male) [c]	Two-sample t-test	-0.01 (-0.22, 0.20)	0.91
Hair style (long)	Two-sample t-test	0.16 (-0.05, 0.36)	0.16
Hearing loss (yes)	Two-sample t-test	-0.05 (-0.45, 0.35))	0.79
Hearing aid user (yes)	Two-sample t test	-0.27 (-0.66, 0.11)	0.27
BCD user (yes) [d]	Two-sample t-test	-0.06 (-0.27, 0.14)	0.52
Quit using BCD (yes)	Two-sample t-test	-0.11 (-0.60, 0.37)	0.64
BCD type (perBCD) [e]	Three-sample t-test	0.20 (-0.74, 1.14)	0.67
Daily hours of BCD use	Kendall's Tau	-0.01 (-0.15, 0.13)	0.90
Single sided deafness (yes)	Two-sample t-test	-0.00 (-0.35, 0.34)	0.99
Outer ear malformation (yes)	Two sample t test	0.58 (0.06, 1.11)	0.03

BCD: bone conduction device; perBCD: percutaneous BCD [a] Based on the statistical test performed, the following statistics are presented: for pearsons r and Kendalls tau correlation coefficients and for t-tests mean difference. All statistics were presented with 95% CI. [b] Bold p-values are statistically significant after Bonferroni correction; [c] For reasons of statistical power, the category 'not specified' was omitted from this analysis. [d] Referring to the group membership as used in the characteristics table; [e] For reasons of statistical power, perBCD was compared to active transcutaneous BCD and the other category combined.
